# Development of genomic evaluation for methane efficiency in Canadian Holsteins*

**DOI:** 10.3168/jdsc.2023-0431

**Published:** 2024-02-01

**Authors:** Hinayah Rojas de Oliveira, Hannah Sweett, Saranya Narayana, Allison Fleming, Saeed Shadpour, Francesca Malchiodi, Janusz Jamrozik, Gerrit Kistemaker, Peter Sullivan, Flavio Schenkel, Dagnachew Hailemariam, Paul Stothard, Graham Plastow, Brian Van Doormaal, Michael Lohuis, Jay Shannon, Christine Baes, Filippo Miglior

**Affiliations:** 1 Lactanet Canada, Guelph, ON, N1K 1E5, Canada; 2 Department of Animal Sciences, Purdue University, West Lafayette, IN 47907; 3 Centre for Genetic Improvement of Livestock, Department of Animal Biosciences, University of Guelph, Guelph, ON, N1G 2W1, Canada; 4 The Semex Alliance, Guelph, ON, N1H 6J2, Canada; 5 Department of Agricultural, Food and Nutritional Science, University of Alberta, Edmonton, AB T6G 2R3, Canada; 6 Institute of Genetics, Department of Clinical Research and Veterinary Public Health, University of Bern, Bern, 3001, Switzerland

## Abstract

Reducing methane (CH_4_) emissions from agriculture, among other sectors, is a key step to reducing global warming. There are many strategies to reduce CH_4_ emissions in ruminant animals, including genetic selection, which yields cumulative and permanent genetic gains over generations. A single-step genomic evaluation for methane efficiency (MEF) was officially implemented in April 2023 for the Canadian Holstein breed, aiming to reduce CH_4_ emissions without affecting production levels. This evaluation was achieved by using milk mid-infrared (MIR) spectral data to predict individual cow CH_4_ production. The genetic evaluation model included milk MIR predicted CH_4_ (CH4_MIR_), along with milk yield (MY), fat yield (FY), and protein yield (PY), as correlated traits. Traits were expressed in kilograms per day (MY, FY, and PY) or grams per day (CH4_MIR_). The MiX99 software was used to fit the single-step, 4-trait animal model. Genomic breeding values for CH4_MIR_ were then obtained by re-parameterization, using recursive genetic linear regression coefficients on MY, FY, and PY, giving a measure of MEF that is genetically independent of the production traits. The estimated breeding values were expressed as relative breeding values with a mean of 100 and standard deviation of 5 for the genetic base population, where a higher value indicates the animal produces lower predicted CH_4_. This national genomic evaluation is another tool that will lower the dairy industry's carbon footprint by reducing CH_4_ emissions without affecting production traits.

## Highlights

• A cow's milk mid-infrared spectra data can be used as a good predictor of its methane production.
• A genomic evaluation for methane efficiency was developed to select for cows that emit less methane.
• Herd owners selecting for methane efficiency can achieve a 20% to 30% reduction in methane emissions from their herd by 2050.

## Graphical Abstract

**Figure:**
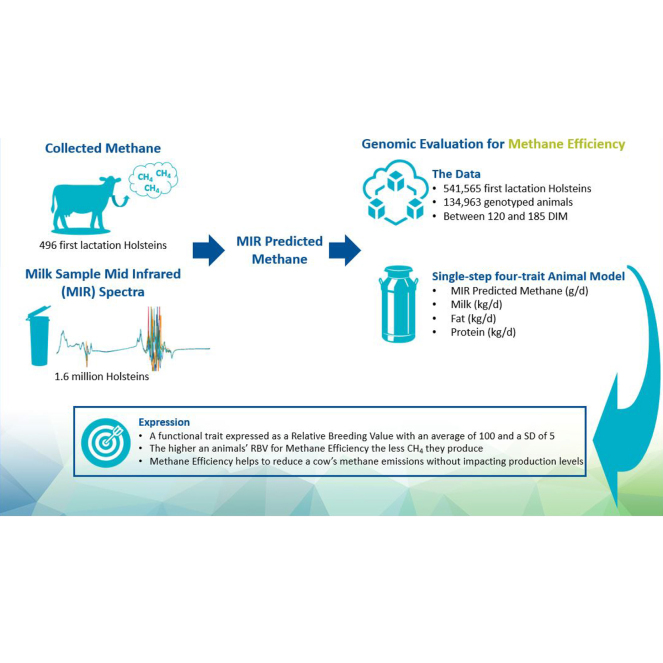
**Summary:** Reducing dairy cattle methane emissions is a key step to limiting global warming and reaching net zero greenhouse gas emissions from dairy farm-level production by 2050. There are many pathways that can be used to reach this industry goal, including genetic selection. We developed a genomic evaluation for methane efficiency using milk mid-infrared spectra as a prediction of methane emissions. This evaluation allows producers to select for cows that emit less methane without affecting milk production.

Concerns about the effects of climate change on environmental sustainability are growing. Increasingly, communities, organizations, and individuals are actively thinking about how they can be more sustainable and create a balanced ecosystem for future generations. This collective effort is reflected in the commitments made by numerous global dairy industry stakeholders, including Dairy Farmers of Canada, to achieve net-zero GHG emissions by 2050. Methane (CH_4_), a potent GHG, which remains in the atmosphere for about 12 yr and makes up 14% of Canada's GHG emissions, has been under the spotlight as it is responsible for almost half the net global temperature change due to human activities in the last decade (Environment and Climate Change Canada, 2022). The goal is to reduce the global CH_4_ production and limit global warming to 1.5°C by 2050 (Global Methane Pledge, 2021; IPCC, 2023).

Even though the agriculture industry is not the sole source of increasing global CH_4_ emissions, it has the potential to mitigate this increase and contribute to climate cooling by reducing its rate of CH_4_ emissions. Looking at the dairy industry specifically and accounting for all on-farm and off-farm GHG involved in the production of 1 kg of milk on Canadian dairy farms, CH_4_ appears as the largest contributor to the milk footprint at close to 50% (Groupe AGECO, 2018). It mostly comes from digestion (enteric fermentation) and, to a lesser extent, manure management. Previous studies have identified that an average Holstein cow produces roughly 426 to 463 g of CH_4_ per day (Denninger et al., 2019; Kamalanathan et al., 2023). Moreover, CH_4_ production is heritable ranging from 0.12 to 0.45 (Lassen and Løvendahl, 2016; Breider et al., 2019; Kamalanathan et al., 2023), which presents an opportunity to decrease CH_4_ emissions by using genetic selection.

Using genetics to select cows with reduced CH_4_ emissions is a permanent and cumulative solution for reducing the dairy sector's GHG emissions. However, measuring CH_4_ is expensive using standard methods (i.e., GreenFeed and sniffers) and difficult, resulting in few animals with recorded CH_4_ (Shetty et al., 2017). Alternative methods, such as the use of mid-infrared (**MIR**) technology, offer cost-effective solutions to predict CH_4_ emissions on a larger scale (Dehareng et al., 2012; Shadpour et al., 2022). The objective of this paper was to present the development and implementation of a routine genomic evaluation system for methane efficiency (**MEF**), launched officially in Canada in April 2023 for the Holstein breed.

Methane production was recorded on 700 Holstein cows from the Ontario Dairy Research Station (Ontario, Canada; 500 cows) and the Dairy Research and Technology Centre (Alberta, Canada; 200 cows) between 2016 and 2022 as part of the Efficient Dairy Genome Project (https://genomedairy.ualberta.ca/) and the Resilient Dairy Genome Project (RDGP, http://www.resilientdairy.ca/). Animal Care Committee approval was obtained from the University of Guelph (animal utilization protocol number: 3503) and from the University of Alberta (animal utilization protocol number: AUP00000170). The data recording at these 2 stations (one GreenFeed per station) is described in Kamalanathan et al. (2023) and Liu et al. (2022). Briefly, first-lactation cows between 120 and 150 DIM at the Ontario Dairy Research Station were moved into a tiestall area. For CH_4_ emission testing, the GreenFeed system (C-Lock Inc., Rapid City, SD) was moved in front of the animal and CH_4_ and carbon dioxide concentrations were measured for roughly 10 min, 4 times a day (0800, 1200, 1600, and 2000 h, from 2016 to 2019) or 3 times a day (0800, 1200, and 1600 h, from 2019 to 2023) for 5 consecutive days. This ensured the sampling of numerous short-term records at different times of the day across multiple days as suggested as the best practice for the GreenFeed system. Methane production data for each day, in grams per day, was the average of the estimated CH_4_ production during the 3 or 4 testing sessions.

At the Dairy Research and Technology Center, CH_4_ emissions were recorded on mixed-parity cows between 3 and 240 DIM housed in a tiestall barn, starting in 2016. Methane measurements were recorded via the GreenFeed system for 12 consecutive days twice per day at 12 h intervals that shifted by an hour each day to cover all 24 h. In 2019, the collection was changed to 3 times per day (0800, 1200, and 1600 h) for 5 consecutive days to better mirror the sampling at the Ontario Dairy Research Station.

Individual milk sample MIR spectra collected routinely in Lactanet laboratories in the provinces of Quebec, Ontario, Alberta (samples from both Alberta and Saskatchewan), and British Columbia were included in the analysis. Roughly 13 million spectra records on 1.6 million Holstein dairy cows from 7,171 herds were collected from 2018 to 2022 using the CombiFoss 7 instruments (Foss Electric A/S, Hillerød, Denmark). Uninformative spectral regions and those related to the high absorption of water were removed, resulting in 241 spectral data points. Those regions remaining for the analysis were roughly 1,000 to 1,550 cm^−1^ (FOSS MIR pins 260 to 402), 1,705 to 1,820 cm^−1^ (FOSS MIR pins 442 to 472), and 2,700 to 2,955 cm^−1^ (FOSS MIR pins 701 to 767). Milk MIR spectra were standardized between laboratories and across time using the approach described by Bonfatti et al. (2017). This methodology uses principal component analysis (**PCA**) to inspect shifts in PCA scores over time to define subsets with homogeneous spectra for the creation of standardization matrices. Principal components for each laboratory were obtained using the prcomp function in R, using data scaled by the scale2 function. All major principal components explaining more than 1% of variance were further investigated to define the changes in patterns over time. For quality control of the spectra within each subset, outliers were detected using Mahalanobis distance and removed based on the chi-squared test statistic (*P* < 0.001). Following the standardization, spectral smoothing pretreatment was applied using a Savitzky-Golay filter with a third-order polynomial and filter width 11 (Savitzky and Golay, 1964). The standardized spectra from all laboratories were combined with test-day production records and only those recorded from first-lactation animals with a calving age of at least 16 mo and between 5 and 305 DIM were retained for further analyses.

Previous analysis showed that the multilayer perceptron artificial neural network based on Bayesian regularization had better prediction performance compared with linear regression models such as PLS regression model, due to the ability to model complex patterns (Shadpour et al., 2022). Therefore, the impact of different input variables was tested using solely this approach. Only those variables available in a complete test-day production record were tested for inclusion as potential input variables along with milk MIR (i.e., milk [**MY**], fat [**FY**], and protein [**PY**] yields, season, lactation, age, and DIM). Ultimately, milk MIR data were used as a sole input variable to have a more flexible prediction to apply to historical data. Following the approach by Shadpour et al. (2022), predictions were performed using the “*brnn*” function available in the R software (Perez Rodriguez and Gianola, 2022), fitting 2-layer neural networks containing 2 neurons. The number of epochs was set to 100 and all other model parameters were set as the default values.

After determining the optimum Bayesian regularized artificial neural network prediction model and the milk MIR input using information from preliminary analysis and Shadpour et al. (2022), a further investigation was done to compare the accuracy of predicting daily CH_4_ production measurements and the accuracy of predicting weekly average CH_4_ production. Weekly averages were calculated based on the week of measurement of each animal. Methane records were matched with the closest test-day milk MIR data. The weekly averages yielded higher accuracies compared with the daily averages and were therefore used for the final model. Only records from first-lactation cows within 5 and 305 DIM with at least 2 daily CH_4_ measurements in the weekly average were included in the analysis and outliers were removed based on the standard deviation (>|3.5| SD). Additionally, a record was required to have a corresponding milk MIR spectrum within 11 d from the middle day of the week measurement. After editing, the dataset used for the prediction included 496 animals, where 96% of cows had their milk MIR data within the same week. Descriptive statistics of the data are shown in Table 1.

**Table 1:** Descriptive statistics for the recorded methane production (n = 496)^1^

Item	Weekly average CH_4_ production (g/d)	Test-day MY (kg/d)	Test-day FY (kg/d)	Test-day PY (kg/d)	AC (mo)	DIM
Mean	490.4	33.3	1.3	1.1	23.8	137.9
SD	81.2	5.0	0.2	0.2	1.5	20.4

1 MY = milk yield; FY = fat yield; PY = protein yield; AC = age at calving.

Prediction accuracy (r) was measured as phenotypic Pearson correlation between recorded and predicted CH_4_. Methane predictions using weekly CH_4_ averages had a prediction accuracy of 0.70. This result was similar to the values previously reported by Shadpour et al. (2022), when using milk MIR data and no milk production data for prediction (i.e., r = 0.57–0.72). Moreover, the root mean square error for the prediction equation was 58.62 g/d. The ability of milk MIR information to accurately predict CH_4_ can be partially explained by the fact that milk MIR spectra include detailed information on milk components including milk fatty acid profiles, which is related to enteric fermentation and CH_4_ emissions (Delfosse et al., 2010; Dijkstra et al., 2011; Dehareng et al., 2012).

Variance components for CH4_MIR_, MY, FY, and PY were estimated using data from the August 2022 data extraction. The final data contained 659,701 records from 462,120 first lactation cows (120–185 DIM) in 5,804 herds. Due to computational demand, 5 different subsets, each representing 10% of the herds from the final dataset, were used for the variance component estimation. On average, subsets contained 64,803 records from 45,137 first-lactation cows in 580 herds. Variance components were estimated for each trait in AIREMLF90 using the AI-REML method (Misztal et al., 2014), with the following 4-trait linear animal model:

**y** = **Xb** + **Z**_1_**htd** + **Z**_2_**a** + **Z**_3_**p** + **e**,

where **y** is a vector of observations for CH4_MIR_, MY, PY, and FY; **b** is a vector of all fixed effects (age at calving, DIM, and year-season of calving); **htd** is a vector of the random HTD effect; **a** is a vector of random animal additive genetic effects; **p** is a vector of random permanent environmental (**PE**) effects; **e** is a vector of random residuals; and **X**, **Z**_1_, **Z**_2_, and **Z**_3_ are the respective incidence matrices. Random effects were assumed to be normally distributed, with means equal to zero. Covariance structure of the 4-trait analysis was as follows:

$$v\left(\begin{array}{c} \begin{array}{c} htd\\ a\\ p \end{array}\\ e \end{array}\right)=\left(\begin{array}{cc} \begin{array}{cc} HTD⊗I & 0\\ 0 & G⊗A \end{array} & \begin{array}{cc} 0 & \;0\\ 0 & \;0 \end{array}\\ \begin{array}{cc} 0\; & 0\\ 0\; & 0 \end{array} & \begin{array}{cc} P⊗I & 0\\ 0 & R⊗I \end{array} \end{array}\right)\;,$$

where **HTD** is the (co)variance (4 × 4) matrix between traits for HTD effects, **G** is the genetic covariance (4 × 4) matrix between traits for animal additive genetic effects, **P** is the (co)variance (4 × 4) matrix between traits due to PE effects, **R** is the residual covariance (4 × 4) matrix between traits, ⊗ is the Kronecker product, **A** is the additive genetic relationship matrix, and **I** is an identity matrix.

Average estimates of genetic parameters from the multitrait analyses are reported in Table 2. Average heritability for CH4_MIR_ was 0.23 (0.01), which was in line with previously reported heritability estimates for CH4_MIR_ (Kandel et al., 2017) and other CH_4_ traits (Lassen and Løvendahl, 2016; Kamalanathan et al., 2023; van Breukelen et al., 2023). Average heritability estimates for MY, FY, and PY were 0.38 (0.01), 0.27 (0.01), and 0.28 (0.01), respectively. These values were in line with those estimated for the official genetic evaluation for these traits (Lactanet, 2021). The CH4_MIR_ had a moderately positive genetic correlation of 0.38 with FY. Kandel et al. (2017) also found a positive genetic correlation with FY that increased from approximately 0.10 to 0.20 across 120 to 180 DIM when using a DIM and milk MIR data in the model to predict daily CH_4_ emissions. Similarly, a positive genetic correlation of 0.21 between FY and CH_4_ production was reported by Pszczola et al. (2019). This means that cows with high FY emit more CH_4_, which can be explained by the positive relationship between milk fatty acids (**MFA**) and CH_4_ production (Dijkstra et al., 2011). Odd- and branched-chain MFA are related to VFA in the rumen (Vlaeminck and Fievez, 2005), which play a role in methanogenesis (Ellis et al., 2008). Moreover, MIR was reported to be a good predictor of MFA (Soyeurt et al., 2011; Fleming et al., 2017), and accordingly, CH4_MIR_ has a positive moderate correlation with FY. Nonetheless, a slight negative correlation of −0.13 and −0.11 was estimated for MY and PY with CH4_MIR_, respectively. Kandel et al. (2017) also found a similar negative genetic correlation with MY around −0.20 and with PY that ranged from approximately −0.18 to −0.08 across 120 to 180 DIM.

**Table 2:** Heritability (diagonal),^1^ genetic correlations (above diagonal),^1^ and phenotypic correlations (below diagonal)^1^

Trait^2^	CH4_MIR_	MY	FY	PY
CH4_MIR_	0.23	−0.13	0.38	−0.11
MY	−0.06	0.38	0.48	0.83
FY	−0.18	0.66	0.27	0.71
PY	0.01	0.90	0.74	0.28

1 Approximated SE < 0.03.

2 CH4_MIR_ = milk mid-infrared predicted methane production; MY = milk yield; FY = fat yield; PY = protein yield.

A multiple-trait single-step genomic evaluation (**ssGBLUP**) was implemented at Lactanet Canada using MiX99 and related software (MiX99 Development Team, 2017). Results presented here refer to data extracted for April 2023 official evaluations, which included 773,743 CH_4MIR_, MY, FY, and PY records from 541,565 primiparous Holsteins between 120 and 185 DIM (daughters of 10,765 sires) from 6,128 herds. Average MY, FY, and PY were 32.5 (SD = 6.2), 1.3 (SD = 0.3), and 1.1 (SD = 0.2) kg/d, respectively. On average, cows produced 492 g of CH4_MIR_ per day (average BW and DMI; 634.9 [SD = 61.9] kg and 20.1 [SD = 2.2] kg/d, respectively), which was slightly higher than previous studies using GreenFeed (Denninger et al., 2019; Kamalanathan et al., 2023). However, this can be explained by the range in DIM used in the current study.

There were 134,963 genotyped animals in the 5 generations of pedigree, including 68,138 cows with data (i.e., CH4_MIR_, MY, FY, and PY records) and genotypes and 7,921 sires of cows with data. Animals were genotyped either with a 50K SNP panel or imputed to 50K using F-Impute (Sargolzaei et al., 2014). The linear animal model for genomic prediction was the same for each of the 4 traits (model specified above). However, in ssGBLUP matrix **A** was replaced by **H** matrix, which combines pedigree and genomic information. Reliability of GEBV was approximated by a weighted (80:20) average of direct genomic value and animal model reliabilities (Sullivan et al., 2005; Sullivan, 2010). Direct genomic value reliabilities were calculated using SNP prediction error co-variances with the SNP-BLUP-REL software (Luke, Finland). Animal model reliabilities were calculated based on effective daughter contributions (Sullivan et al., 2005).

Methane efficiency was defined as genetic residual CH_4_ production, or CH_4_ genetically independent of MY, FY, and PY, and derived using a recursive model operational tool (Jamrozik et al., 2017, 2021), and the scale of the final MEF is then reversed. As expected, MEF had no genetic correlation with MY, PY, or FY, meaning selection for MEF will have no impact on milk production. Reliabilities of GEBV for MEF, being a linear function of 4 traits, were approximated by a selection index method (Sullivan et al., 2005).

To maintain consistency with other Canadian genetic evaluations, MEF was expressed as a relative breeding value (**RBV**), with a mean of 100 and a standard deviation of 5 for base bulls (born 2008–2017, with an official MEF evaluation). The higher values indicate a higher (more desirable) MEF for an animal. Therefore, the higher a sire's RBV for MEF, the less CH_4_ their daughters are expected to produce. Moreover, predicted and recorded CH_4_ emissions were compared for low (<−1SD), medium (−1 ≥ SD ≤ +1), and high (>+1SD) RBV classes for MEF (Figure 1). On average, cows in the lowest RBV class (<95; 72 cows) had the highest recorded CH_4_ emissions at 518.8 g/d and CH4_MIR_ at 521.0 g/d. Cows with RBV between 95 and 105 (355 cows) had recorded CH_4_ and CH4_MIR_ emissions of 492.8 and 489.7 g/d, respectively. Last, cows with the highest RBV for MEF (>105; 44 cows) had average recorded CH_4_ and CH4_MIR_ emissions of 464.0 and 465.8 g/d, indicating that selecting animals with high MEF evaluations will lead to a reduction in CH_4_ emissions.

**Figure 1:**
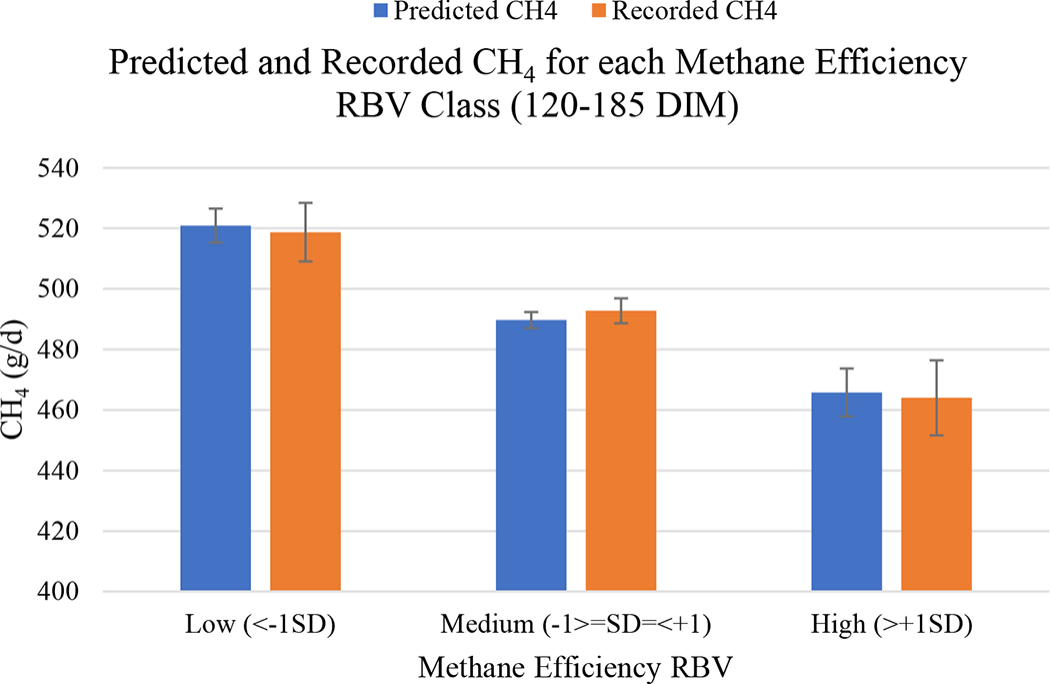
Mid-infrared predicted CH_4_ and recorded CH_4_ (g/d) for low, medium, and high classes (120–185 DIM) of methane efficiency relative breeding values (RBV; n = 471). Error bars represent SE. Predicted CH_4_ (blue): low = 5.6, medium = 2.7, high = 7.9. Recorded CH_4_ (orange): low = 9.7, medium = 4.1, high = 12.4.

The average reliability of RBV for MEF of the 68,138 genotyped cows was 86.7% (SD = 1.5%; ranging from 68% to 95%), whereas the average reliability for the 473,427 nongenotyped cows was 56.3% (SD = 3.6%). There are 2,142 Holstein sires with an official evaluation status for MEF, with an average reliability of 95.9% (SD = 2.5%) ranging from 72% to 99%. To have an official evaluation, a sire is required to have 20 daughters from 5 herds and a minimum reliability of 70%. Daughter averages for CH4_MIR_ were regressed on sires' RBV for MEF to translate RBV to an equivalent expected reduction in CH_4_ for their daughters. Daughters of bulls with a breed average 100 RBV for MEF produced on average 486.5 g/d of CH4_MIR_. A 5-point RBV increase for MEF (1 SD) has the expected effect of decreasing CH4_MIR_ in daughters by 7.55 g/d, or 3 kg per year, which is approximately a 1.5% reduction in CH_4_ emissions per cow per year. Overall, herd owners consistently selecting animals with RBV for MEF of 105 or greater can achieve a 20% to 30% reduction in methane emissions from their herd by 2050.

To estimate proof correlations between MEF and the 2 selection indexes (lifetime performance index [LPI] and profit-based genetic selection index [Pro$]) as well as other routinely evaluated traits in Canada, 2,142 genotyped Holstein bulls with official evaluations for MEF were selected. Methane efficiency proofs were uncorrelated with LPI, Pro$, and the production traits (milk, fat, and protein). Slightly positive and favorable correlations of 0.17 and 0.23 were identified with the health and fertility component of LPI and metabolic disease resistance, respectively. In addition, MEF did not have a significant correlation with feed efficiency (proof correlation of −0.14). This means that selection for methane efficiency will not affect feed efficiency, which is a trait calculated to be genetically independent of ECM and metabolic BW.

Some limitations in this study should be noted. The prediction of methane using milk MIR data can be further refined, as the current model is based on a limited sample size from 2 research stations. The plan is to enlarge the quantity of data used for the prediction as new records become available and to validate the prediction formally using an external data source, independent of the dataset used for model training. Nevertheless, the prediction is accurate enough to start genomic selection for this important new trait in Canada and move the dairy cattle population in the right direction by reducing CH_4_ emissions. The strict protocol of CH_4_ collection, the focus on using only mid-lactation first-parity Holsteins, and the robust and homogeneous milk MIR data recording and storage across Lactanet laboratories in Canada are all elements that provide a sound technical decision for the launch of genomic evaluation for methane efficiency.

Predicting average daily CH_4_ production using milk MIR was proven to be a key and rapid alternative to direct CH_4_ measurements. Predicted CH_4_ using milk MIR data led to the development of routine genomic evaluations for MEF that were officially implemented in April 2023 for the Holstein breed in Canada, focusing on selection for reduced CH_4_ emissions without affecting milk, fat, and protein production levels. Methane efficiency evaluations are a new tool to help reduce the dairy industry's environmental footprint and contribute to the goal of reaching net zero GHG emissions by 2050 without affecting milk production.

## References

[bib1] Bonfatti V., Fleming A., Koeck A., Miglior F. (2017). Standardization of milk infrared spectra for the retroactive application of calibration models. J. Dairy Sci..

[bib2] Breider I.S., Wall E., Garnsworthy P.C. (2019). Short communication: Heritability of methane production and genetic correlations with milk yield and body weight in Holstein-Friesian dairy cows. J. Dairy Sci..

[bib3] Dehareng F., Delfosse C., Froidmont E., Soyeurt H., Martin C., Gengler N., Vanlierde A., Dardenne P. (2012). Potential use of milk mid-infrared spectra to predict individual methane emission of dairy cows. Animal.

[bib4] Delfosse O., Froidmont E., Fernandez Pierna J.A., Martin C., Dehareng F. (2010). Proceedings of the 4th International Conference on Greenhouse Gases and Animal Agriculture. Banff, Canada.

[bib5] Denninger T.M., Dohme-Meier F., Eggerschwiler L., Vanlierde A., Grandl F., Gredler B., Kreuzer M., Schwarm A., Münger A. (2019). Persistence of differences between dairy cows categorized as low or high methane emitters, as estimated from milk mid-infrared spectra and measured by GreenFeed. J. Dairy Sci..

[bib6] Dijkstra J., van Zijderveld S.M., Apajalahti J.A., Bannink A., Gerrits W.J.J., Newbold J.R., Perdok H.B., Berends H. (2011). Relationships between methane production and milk fatty acid profiles in dairy cattle. Anim. Feed Sci. Technol..

[bib7] Ellis J.L., Dijkstra J., Kebreab E., Bannink A., Odongo N.E., McBride B.W., France J. (2008). Aspects of rumen microbiology central to mechanistic modelling of methane production in cattle. J. Agric. Sci..

[bib8] Environment and Climate Change Canada (2022). National Inventory Report 1990–2020: Greenhouse Gas Sources and Sinks in Canada. https://publications.gc.ca/collections/collection_2022/eccc/En81-4-2020-1-eng.pdf.

[bib9] Fleming A., Schenkel F.S., Chen J., Malchiodi F., Bonfatti V., Ali R.A., Mallard B., Corredig M., Miglior F. (2017). Prediction of milk fatty acid content with mid-infrared spectroscopy in Canadian dairy cattle using differently distributed model development sets. J. Dairy Sci..

[bib10] Global Methane Pledge (2021). European Commission, United States of America. Global Methane Pledge. https://www.globalmethanepledge.org/.

[bib11] Groupe AGECO (2018). Environmental life cycle assessment of Canadian milk production. 2016 data and results update. Executive Summary. https://www.dairyfarmers.ca/content/download/6327/56092/version/2/file/LCA__ExecutiveSummary.pdf.

[bib12] IPCC (Intergovernmental Panel on Climate Change) (2023). Climate Change 2021–The Physical Science Basis: Working Group I Contribution to the Sixth Assessment Report of the Intergovernmental Panel on Climate Change.

[bib13] Jamrozik J., Johnston J., Sullivan P.G., Miglior F. (2017). Recursive model approach to traits defined as ratios: Genetic parameters and breeding values. J. Dairy Sci..

[bib14] Jamrozik J., Kistemaker G.J., Sullivan P.G., Van Doormaal B.J., Chud T.C.S., Baes C.F., Schenkel F.S., Miglior F. (2021). Genomic evaluation for feed efficiency in Canadian Holsteins. Interbull Bull..

[bib15] Kamalanathan S., Houlahan K., Miglior F., Chud T.C.S., Seymour D.J., Hailemariam D., Plastow G., de Oliveira H.R., Baes C.F., Schenkel F.S. (2023). Genetic analysis of methane emission traits in Holstein dairy cattle. Animals (Basel).

[bib16] Kandel P.B., Vanrobays M.-L., Vanlierde A., Dehareng F., Froidmont E., Gengler N., Soyeurt H. (2017). Genetic parameters of mid-infrared methane predictions and their relationships with milk production traits in Holstein cattle. J. Dairy Sci..

[bib17] Lactanet (2021). Heritability estimates used for genetic evaluation in Canada. https://lactanet.ca/en/heritability-estimates-used-for-genetic-evaluation-in-canada/.

[bib18] Lassen J., Løvendahl P. (2016). Heritability estimates for enteric methane emissions from Holstein cattle measured using noninvasive methods. J. Dairy Sci..

[bib19] Liu R., Hailemariam D., Yang T., Miglior F., Schenkel F., Wang Z., Stothard P., Zhang S., Plastow G. (2022). Predicting enteric methane emission in lactating Holsteins based on reference methane data collected by the GreenFeed system. Animal.

[bib20] MiX99 Development Team (2017). http://www.luke.fi/mix99.

[bib21] Misztal I., Tsuruta S., Lourenco D.A.L., Masuda Y., Aguilar I., Legarra A., Vitezica Z. (2014). http://nce.ads.uga.edu/wiki/lib/exe/fetch.php?media=blupf90_all4.pdf.

[bib22] Perez Rodriguez P., Gianola D. (2022). brnn: Bayesian Regularization for Feed-Forward Neural Networks. R package version 0.9.2. https://CRAN.R-project.org/package=brnn.

[bib23] Pszczola M., Calus M.P.L., Strabel T. (2019). Short communication: Genetic correlations between methane and milk production, conformation, and functional traits. J. Dairy Sci..

[bib24] Sargolzaei M., Chesnais J.P., Schenkel F.S. (2014). A new approach for efficient genotype imputation using information from relatives. BMC Genomics.

[bib25] Savitzky A., Golay M.J.E. (1964). Smoothing and differentiation of data by simplified least squares procedures. Anal. Chem..

[bib26] Shadpour S., Chud T.C., Hailemariam D., Plastow G., Oliveira H.R., Stothard P., Lassen J., Miglior F., Baes C.F., Tulpan D., Schenkel F.S. (2022). Predicting methane emission in Canadian Holstein dairy cattle using milk mid-infrared reflectance spectroscopy and other commonly available predictors via artificial neural networks. J. Dairy Sci..

[bib27] Shetty N., Difford G., Lassen J., Løvendahl P., Buitenhuis A.J. (2017). Predicting methane emissions of lactating Danish Holstein cows using Fourier transform mid-infrared spectroscopy of milk. J. Dairy Sci..

[bib28] Soyeurt H., Dehareng F., Gengler N., McParland S., Wall E., Berry D.P., Coffey M., Dardenne P. (2011). Mid-infrared prediction of bovine milk fatty acids across multiple breeds, production systems, and countries. J. Dairy Sci..

[bib29] Sullivan P.G. (2010).

[bib30] Sullivan, P. G., F. Miglior, and G. J. Kistemaker. 2005. Approximate reliability of an index of estimated breed values. Research Report to the Interbull Technical Committee, Uppsala, Sweden.

[bib31] van Breukelen A.E., Aldridge M.N., Veerkamp R.F., Koning L., Sebek L.B., de Haas Y. (2023). Heritability and genetic correlations between enteric methane production and concentration recorded by GreenFeed and sniffers on dairy cows. J. Dairy Sci..

[bib32] Vlaeminck B., Fievez V. (2005). Milk odd and branched-chain fatty acids to predict ruminal methanogenesis in dairy cows. Commun. Agric. Appl. Biol. Sci..

